# Deep Convergence, Shared Ancestry, and Evolutionary Novelty in the Genetic Architecture of *Heliconius* Mimicry

**DOI:** 10.1534/genetics.120.303611

**Published:** 2020-09-03

**Authors:** Jake Morris, Joseph J. Hanly, Simon H. Martin, Steven M. Van Belleghem, Camilo Salazar, Chris D. Jiggins, Kanchon K. Dasmahapatra

**Affiliations:** * Department of Biology, University of York, Heslington YO10 5DD, United Kingdom; † Department of Zoology, University of Cambridge, Downing Street, Cambridge CB2 3EJ, United Kingdom; ‡ Institute of Evolutionary Biology, The University of Edinburgh, Ashworth Laboratories, Edinburgh EH9 3FL, United Kingdom; § Biology Program, Faculty of Natural Sciences, Universidad del Rosario, Bogotá 111221, Colombia

**Keywords:** adaptation, *cis*-regulation, collateral evolution, genetic architecture

## Abstract

Convergent evolution can occur through different genetic mechanisms in different species. It is now clear that convergence at the genetic level is also widespread, and can be caused by either (i) parallel genetic evolution, where independently evolved convergent mutations arise in different populations or species, or (ii) collateral evolution in which shared ancestry results from either ancestral polymorphism or introgression among taxa. The adaptive radiation of *Heliconius* butterflies shows color pattern variation within species, as well as mimetic convergence between species. Using comparisons from across multiple hybrid zones, we use signals of shared ancestry to identify and refine multiple putative regulatory elements in *Heliconius melpomene* and its comimics, *Heliconius elevatus* and *Heliconius besckei*, around three known major color patterning genes: *optix*, *WntA*, and *cortex*. While we find that convergence between *H. melpomene* and *H. elevatus* is caused by a complex history of collateral evolution via introgression in the Amazon, convergence between these species in the Guianas appears to have evolved independently. Thus, we find adaptive convergent genetic evolution to be a key driver of regulatory changes that lead to rapid phenotypic changes. Furthermore, we uncover evidence of parallel genetic evolution at some loci around *optix* and *WntA* in *H. melpomene* and its distant comimic *Heliconius erato*. Ultimately, we show that all three of convergence, conservation, and novelty underlie the modular architecture of *Heliconius* color pattern mimicry.

## Abstract (precis)

Phenotypic convergence between taxa can be caused by divergent genetic evolution (different genetic pathways), parallel genetic evolution (convergent mutations), or collateral evolution (shared ancestry). *Heliconius* butterflies have bright mimetic color patterns shared between multiple species, making an excellent .....

CONVERGENT evolution is a natural experiment in repeated evolution of similar traits, and offers unique insights into the evolutionary process (Blount *et al.* 2018). It is widespread across the tree of life, critical to the composition of ecosystems (Sage *et al.* 2012) (*e.g.*, the repeated colonization of land/water/air by different taxonomic groups), and underpins the ability of organisms to exploit novel environments (*e.g.*, the repeated evolution of drug/insecticide/drought resistance (Farhat *et al.* 2013). The genetic changes causing convergence can be categorized as (i) divergent genetic mechanisms, (ii) parallel genetic evolution, or (iii) collateral evolution (Stern 2013). With divergent genetic mechanisms, different loci cause the same phenotype in different lineages. In parallel genetic evolution, different alleles at the same locus cause trait convergence (this includes cases where the same mutation has arisen multiple times) (Tishkoff *et al.* 2007), whereas in collateral evolution, convergence results from the sharing of alleles that are identical by descent, either because the alleles were present in an ancestral population (Jones *et al.* 2012), or from the introgression of alleles from one species/taxon to another (Huerta-Sánchez *et al.* 2014).

*Cis*-regulatory evolution has been implicated in several examples of convergent evolution in vertebrates (Booker *et al.* 2016; Partha *et al.* 2017; Tollis *et al.* 2018; Feigin *et al.* 2019), suggesting that trait evolution proceeding via *cis*-regulatory changes to conserved regulatory pathways may be recurrent and predictable. *Cis*-regulatory evolution is a powerful mechanism that can result in rapid developmental and physiological changes (Wittkopp and Kalay 2012). This is because multiple enhancers at the same gene can each control the gene’s expression in different cell types or developmental times. In these cases, the modular architecture can isolate the effects of a mutation to a single trait (Feigin *et al.* 2019), circumventing the pleiotropic effects that might constrain adaptive evolution in the protein-coding sequence. Notable examples in which modular enhancers drive convergent evolution include the gain of melanic wing spots in *Drosophila elegans* and *Drosophila tristis* through enhancers of the gene *yellow* (Prud’homme *et al.* 2006), coat coloration phenotypes via regulation of the gene *Agouti* in *Peromyscus* mice (Steiner *et al.* 2007; Linnen *et al.* 2009, 2013), loss of *Drosophila* larval trichomes through mutations to regulatory regions of *ovo*/*svb* (Frankel *et al.* 2012), and pelvic reduction in sticklebacks due to enhancer deletions at the gene *pitx1* (Chan *et al.* 2010). A modular *cis*-regulatory architecture has also been proposed as a flexible toolkit controlling wing color patterning in *Heliconius* butterflies (Wallbank *et al.* 2016; Van Belleghem *et al.* 2017).

Müllerian mimicry is ubiquitous among neotropical *Heliconius* butterflies, with multiple species evolving convergent, bright, aposematic wing color patterns. At the same time, in several species such as *Heliconius erato* and *Heliconius melpomene*, phenotypic divergence within species is also present in the form of geographic color pattern races (Figure 1), with color pattern loci easily identifiable in population genomic studies across hybrid zones as clear islands of divergence in the genome (Baxter *et al.* 2010; Counterman *et al.* 2010; Nadeau *et al.* 2013, 2014). Owing to the presence of repeated color pattern phenotypes, *Heliconius* butterflies are an excellent system for studying the genetic basis of convergent evolution (Baxter *et al.* 2008; Merrill *et al.* 2015). The small number of mimicry genes controlling the majority of color pattern elements, have been identified across *Heliconius* species using a combination of QTL mapping, genome-wide association studies across color pattern hybrid zones, and gene expression studies. Across multiple species, parallel genetic evolution at the genes *optix*, *cortex*, and *WntA* is known to control red-orange pattern elements (Baxter *et al.* 2008; Reed *et al.* 2011; Martin *et al.* 2014; Huber *et al.* 2015; Lewis *et al.* 2019), white and yellow pattern elements (Nadeau *et al.* 2016), and melanic patterning (Martin *et al.* 2012; Gallant *et al.* 2014; Mazo-Vargas *et al.* 2017; Moest *et al.* 2019; Morris *et al.* 2019; Van Belleghem *et al.* 2020), respectively.

**Figure 1:**
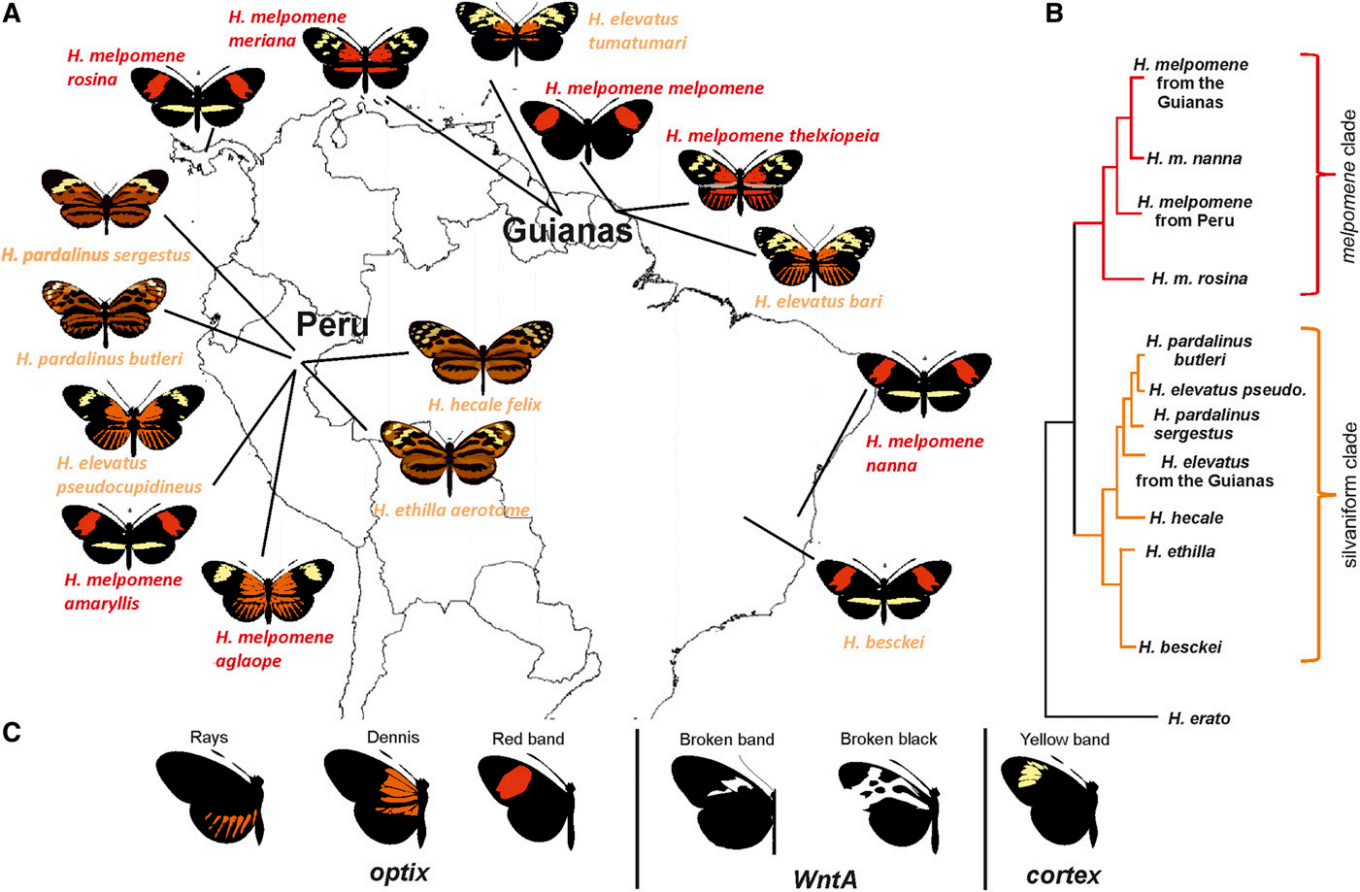
(A) Map showing geographic distribution of color pattern races of the silvaniform clade species (orange; *H. pardalinus*, *H. elevatus*, *H. besckei*, *H. ethilla*, and *H. hecale*) and *H. melpomene* (red) used in the analyses. The postman color pattern is found in *H. melpomene amaryllis*/*nanna*/*rosina*/*melpomene* and *H. besckei* (*H. m. melponene* lacks the yellow hindwing bar). The dennis-rayed pattern is found in *H. melpomene aglaope*/*thelxiopeia*/*meriana* and *H. elevatus pseudocupidineus*/*bari*/*tumatumari* (*H. m. meriana* and *H. e. tumatumari* lack hindwing rays). (B) Cladogram showing the relationships between taxa, based on the species topology inferred here. Note the paraphyly of the species *H. elevatus* and *H. pardalinus* (Dasmahapatra *et al.* 2012). The species *H. erato*, used here as an outgroup, mimics the appearance of the races of *H. melpomene* with which it co-occurs. (C) Phenotypes investigated in this study and known to be controlled by the three major wing patterning genes, *optix*, *cortex*, and *WntA*.

Multiple putative regulatory elements have now been identified in *H. erato* around all three major wing patterning genes (*optix*, *cortex*, and *WntA*) using comparisons between phenotypically distinct races across multiple hybrid zones (Van Belleghem *et al.* 2017). In the *H. melpomene* clade the picture is less complete. ABBA-BABA comparisons and changes in phylogenetic topologies have shown that mimetic resemblance between some races of several *H. melpomene*-silvaniform clade species (*H. melpomene*, *H. elevatus*, *H. timareta*, and *H. besckei*; Figure 1B) are the result of collateral evolution, via the introgression of color pattern alleles at *optix* and *cortex* among the species (Dasmahapatra *et al.* 2012; Pardo-Diaz *et al.* 2012; Zhang *et al.* 2016). Association mapping across a number of *H. melpomene* and *H. timareta* taxa in conjunction with recombination breakpoint analysis (which included *H. elevatus*), was used to define both a 25 kb and an 11 kb regulatory element at *optix* associated with the presence and absence of the red hindwing rays and the forewing dennis phenotypes, respectively (Wallbank *et al.* 2016). However, these genomic regions are still relatively large, and no regulatory element for red band has yet been found. Similarly, around *cortex* it has also been shown that introgression between races of *H. melpomene* and *H. cydno* has likely allowed these species to share variation in the hindwing yellow bar phenotype (Enciso-Romero *et al.* 2017). However, no yellow band regulatory element has been identified at *cortex*. Furthermore, patterns of introgression and any regulatory elements around *WntA* are thus far unknown in the *H. melpomene*-silvaniform clade.

In this study, we investigate the contributions of three genetic modes of evolution, divergent genetic mechanisms, parallel genetic evolution, and collateral evolution to explain the remarkable convergent wing color pattern phenotypes found in *Heliconius* butterflies. In particular, we use phylogenetic analysis across multiple hybrid zone comparisons to delimit narrow regions of the genome associated with color pattern elements due to shared ancestry (either from introgression or ancestral polymorphism). We do this by identifying genomic regions that show genotype-by-phenotype associations and particular phylogenetic histories consistent with controlling specific wing color pattern phenotypes. This allows us for the first time to look at the mechanism of convergence between Guianese *H. melpomene* and *H. elevatus*. We propose that these narrow regions are putative modular regulatory elements, with each controlling a specific wing pattern phenotype. We identify these around all three of the major wing patterning genes; *optix*, *cortex*, and *WntA*, in *H. melpomene* and its silvaniform mimics and determine the ancestral origins of each element. Finally, we investigate the homology and conservation of these regulatory elements between *H. melpomene* and *H. erato*, as well as across other Lepidoptera species.

## Materials and Methods

### Sample collection and sequencing

We used whole genome sequences of 53 individuals representing six species (*H. melpomene*, *H. elevatus*, *H. besckei*, *H. pardalinus*, *H. ethilla*, and *H. hecale*) and 15 races (Supplemental Materials, Table S1) from two hybrid zones in Peru and the Guianas, as well as two taxa from the Eastern Amazon in Brazil (*H. m. nanna* and *H. besckei*) (Figure 1). This includes data from newly sequenced samples of two *H. elevatus tumatumari*, two *H. pardalinus butleri*, and one *H. elevatus bari*. For these new samples, RNA-free genomic DNA was extracted from thoracic tissue using a Qiagen DNeasy Blood and Tissue Kit. Libraries were prepared using Illumina TruSeq DNA PCR-Free Library Preparation Kits with an insert size of ∼350 bp. Libraries were 100 or 125 bp pair-end sequenced to 30–40× coverage on an Illumina HiSeq 2500 instrument at the FAS Center for Systems Biology, Harvard (ENA accession number PRJEB37067).

### Variant calling

We aligned sequences to the *H. melpomene* reference genome v2 (Davey *et al.* 2016; LepBase http://ensembl.lepbase.org) using BWA MEM (Li and Durbin 2009). We then sorted BAM files using Samtools (Li *et al.* 2009) and marked duplicate reads using PicardTools (http://broadinstitute.github.io/picard/). We then called genotypes in gVCF format with GATK’s HaplotypeCaller with the parameters -baq CALCULATE_AS_NECESSARY, -hets 0.02 and-emitRefConfidence GVCF, -gt_mode DISCOVERY and–dontUseSoftClippedBases. Subsequently we combined GVCFs before genotyping them, using CombineGVCFs and GenotypeGVCFs respectively (Van der Auwera *et al.* 2013). Genotypes were then marked as missing (N) with Bcftools v1.3.1 if minimum read depth was <5 or GQ <20, while sites with a minor allele frequency lower than 2/53 across all samples were removed. Python scripts (available at https://github.com/simonhmartin) were used to parse variant call formats (VCFs) to prepare files for use in phylogenetic weighting analyses. SNPs with >10% missing data across taxa were removed.

### Identification of candidate regulatory modules based on shared ancestry

Three major loci (*cortex*, *optix*, and *WntA*) control the main color pattern differences between the postman and dennis-rayed races of *H. melpomene* we examine here (Figure 1; Baxter *et al.* 2010). Previous studies have shown that mimicry between *H. melpomene* races and closely related silvaniform species such as *H. elevatus* and *H. besckei* is a consequence of shared alleles (Dasmahapatra *et al.* 2012; Zhang *et al.* 2016). Therefore, the signal of shared ancestry between comimics can be used to identify narrow genomic intervals that may control color pattern elements (Wallbank *et al.* 2016; Van Belleghem *et al.* 2017). We look for these patterns of shared ancestry around *cortex*, *optix*, and *WntA*. Where previous studies have delimited intervals (Wallbank *et al.* 2016), we constrain our search to these regions.

In order to identify regions that show shared ancestry among different comimetic species at these loci, we employed a descriptive phylogenetic weighting method called Topology Weighting by Iterative Sampling of Subtrees (Twisst; Martin and Van Belleghem 2017). This method provides a quantitative summary of a tree by weighting different subtree topologies according to their occurrence within the tree. Each of our Twisst comparisons used six taxa. We first used RAxML v8.2.4 (Stamatakis 2014) with model -GTRCAT to build maximum-likelihood trees for 100 SNP sliding windows (slide every 25 SNPs) across the entirety of chromosomes 10, 15, and 18 (which contain the major color pattern loci *WntA*, *cortex*, and *optix*, respectively). Trees were built only for windows where all samples had ≥30 SNPs. We used a dynamic threshold as implemented by Twisst to estimate weightings in all analyses, such that trees were sampled until the 95% binomial confidence interval around each weighting was <5%.

For each six taxon Twisst comparison there are 105 potential tree topologies, with just five topologies for each phenotype clearly indicative of shared ancestry related to phenotype (see Figures S2–S6). These trees show monophyly of the nonsister taxa with convergent pattern elements, while the other four taxa group as expected based on the species tree. A topology weighting of 0 indicates that none of the trees for that genomic window were of these five topologies, while a topology weighting of +1 indicates that all of the trees were among these five topologies. We used these six taxon Twisst comparisons to identify putative regions controlling wing pattern elements across three geographic contexts: Peruvian hybrid zone, Guianese hybrid zone, and *H. besckei-H. melpomene* taxa. These geographically distant comparisons allow semi-independent inferences about genetic elements controlling six wing pattern phenotypes; hindwing red/orange “rays,” forewing proximal red/orange “dennis” patch, and forewing red “band” controlled by *optix* expression, broken black and broken band forewing variation controlled by *WntA* expression, and the yellow forewing band controlled by *cortex* expression (Figure 1C).

### Taxon selection for Twisst comparisons

**Figure 2:**
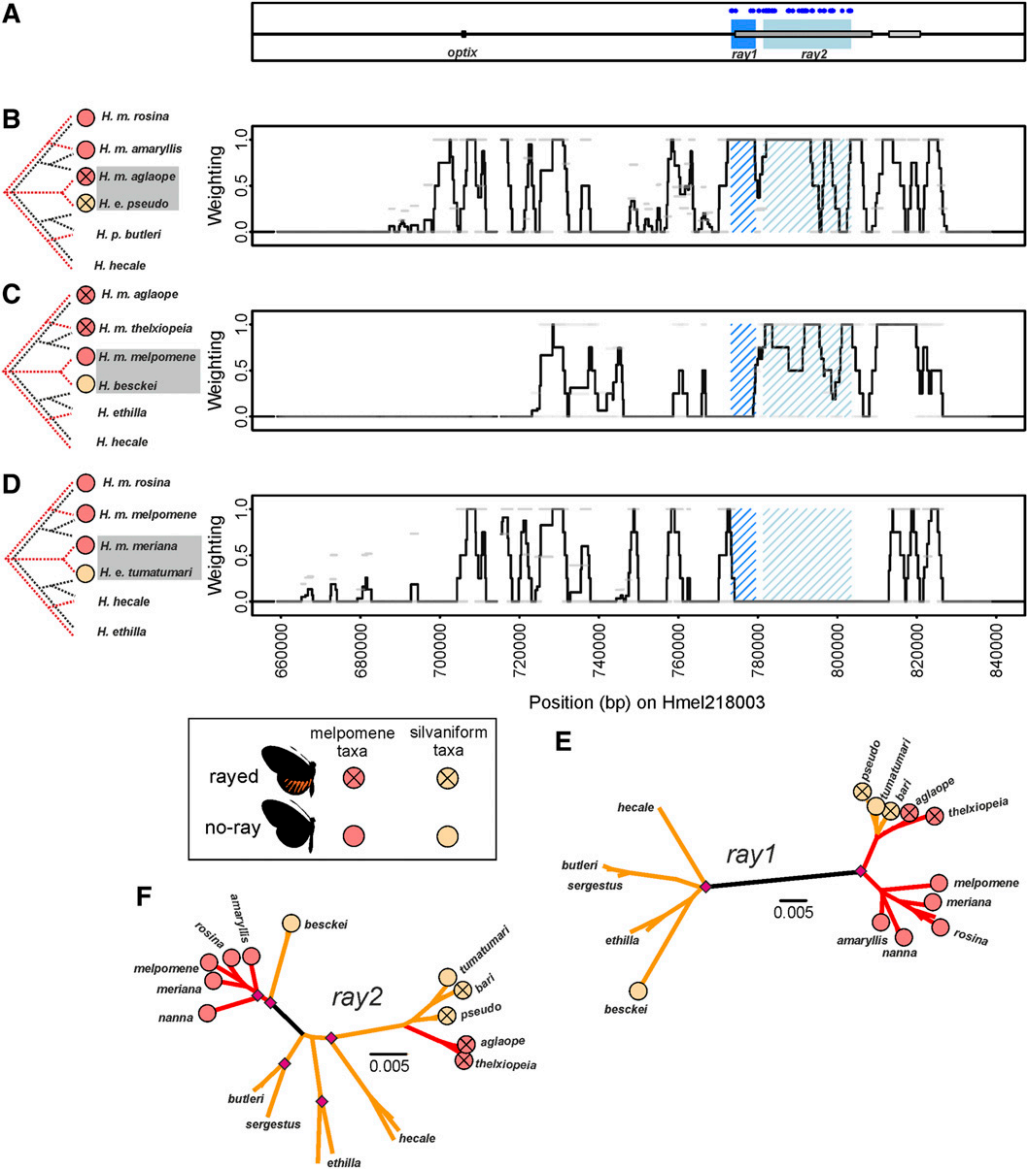
Twisst comparisons across the *optix* region of scaffold Hmel218003. (A) Location of *optix* and *ray* elements. Dark blue (*ray1*) and light blue (*ray2*) shading shows putative functional elements. Gray boxes show ray and dennis regulatory elements respectively as previously delimited in Wallbank *et al.* (2016). Diagnostic fixed SNPs between phenotypes shown with blue dots in (A). Twisst comparison (100 SNP windows sliding by 25 SNPs) using (B) Peruvian hybrid zone taxa, (C) *H. besckei* and Guianese hybrid zone *H. melpomene*, and (D) Guianese hybrid zone taxa. Black trees to left show species topology, while red trees shows groupings (taxa shaded in gray) that indicate shared ancestry between the heterospecific mimetic taxa. Weighting (black line) is the mean from all four overlapping windows for that region, light gray bars show weighting for each 100 SNP window. A weighting of +1 means 100% of trees at that genomic interval show shared ancestry between the heterospecific mimetic taxa. Mimetic phenotypes for taxa are shown by circles; red circles for *H. melpomene* clade and orange circles for silvaniform taxa. (E and F) Maximum likelihood phylogenies of the *ray* elements with red branches joining *H. melpomene* taxa and orange branches joining silvaniform taxa. Node bootstrap support; pink diamonds ≥ 95%, green diamonds 75–94%. Black branch (illustrative only) separates the silvaniform and *H. melpomene* clades (excluding those taxa where introgression appears to have occurred).

**Figure 3:**
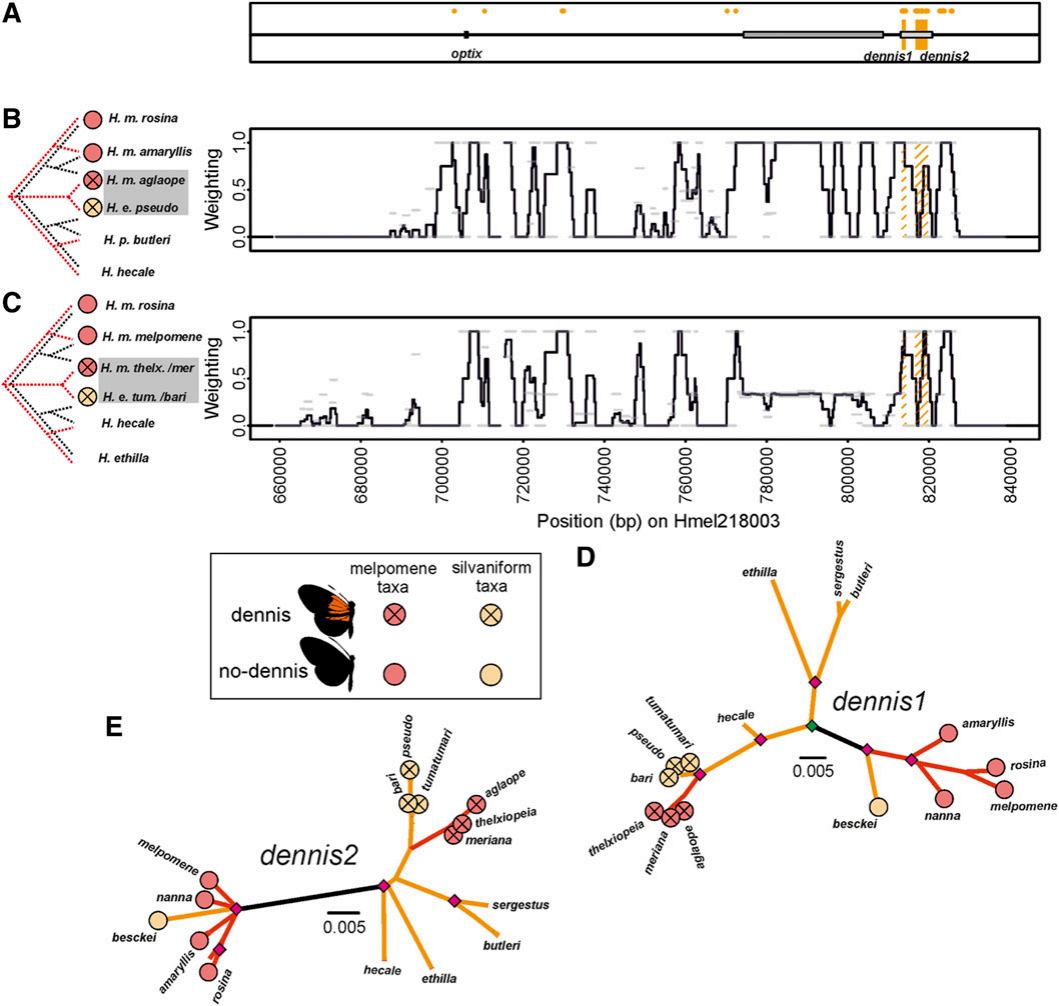
Twisst comparisons across the *optix* region of scaffold Hmel218003. (A) Location of *optix* and *dennis* elements. Gray boxes show ray and dennis regulatory elements respectively as previously delimited in Wallbank *et al.* (2016). Orange shading shows putative functional elements, *dennis1* and *dennis2*. Diagnostic fixed SNPS between phenotypes shown with orange dots in (A). Twisst comparison (100 SNP windows sliding by 25 SNPs) using (B) Peruvian hybrid zone taxa; (C) Guianese hybrid zone taxa. Mimetic phenotypes for taxa are shown by circles; red circles for *H. melpomene* clade and orange circles for silvaniform taxa. (D and E) Maximum likelihood phylogenies of *dennis* elements; see Figure 2 legend for more detailed explanations. Note position of black branch in *dennis2* phylogeny, which has been drawn on the longest branch from where *H. besckei* connects to phylogeny.

**Figure 4:**
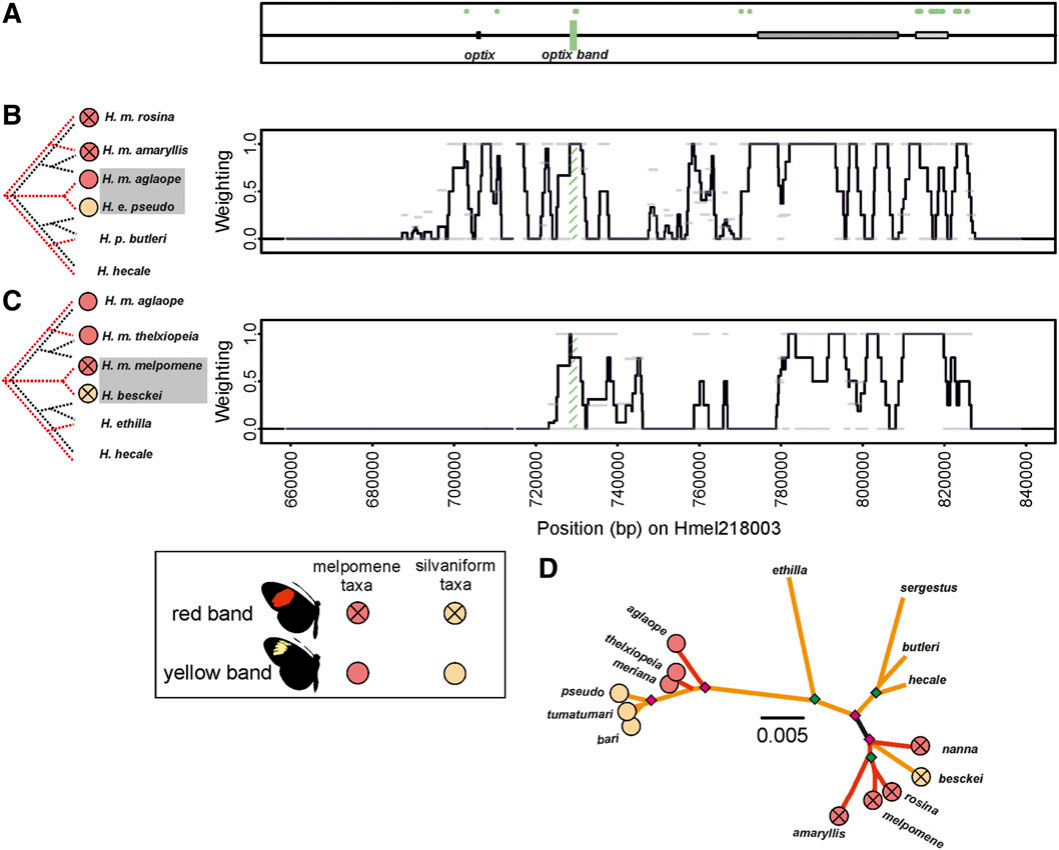
Twisst comparisons across the *optix* region of scaffold Hmel218003. (A) Shows location of *optix* and putative *optix band* element. Gray boxes show ray and dennis regulatory elements respectively as previously delimited in Wallbank *et al.* (2016). Green shading shows putative functional elements. Diagnostic fixed SNPS between phenotypes shown with green dots in (A). Twisst comparison (100 SNP windows sliding by 25 SNPs) using (B) Peruvian hybrid zone taxa, and (C) *H. besckei* and Guianese hybrid zone taxa. Mimetic phenotypes for taxa are shown by circles; red circles for *H. melpomene* clade and orange circles for silvaniform taxa. (D) Maximum likelihood phylogeny of putative *optix band* element; see Figure 2 legend for more detailed explanations.

**Figure 5:**
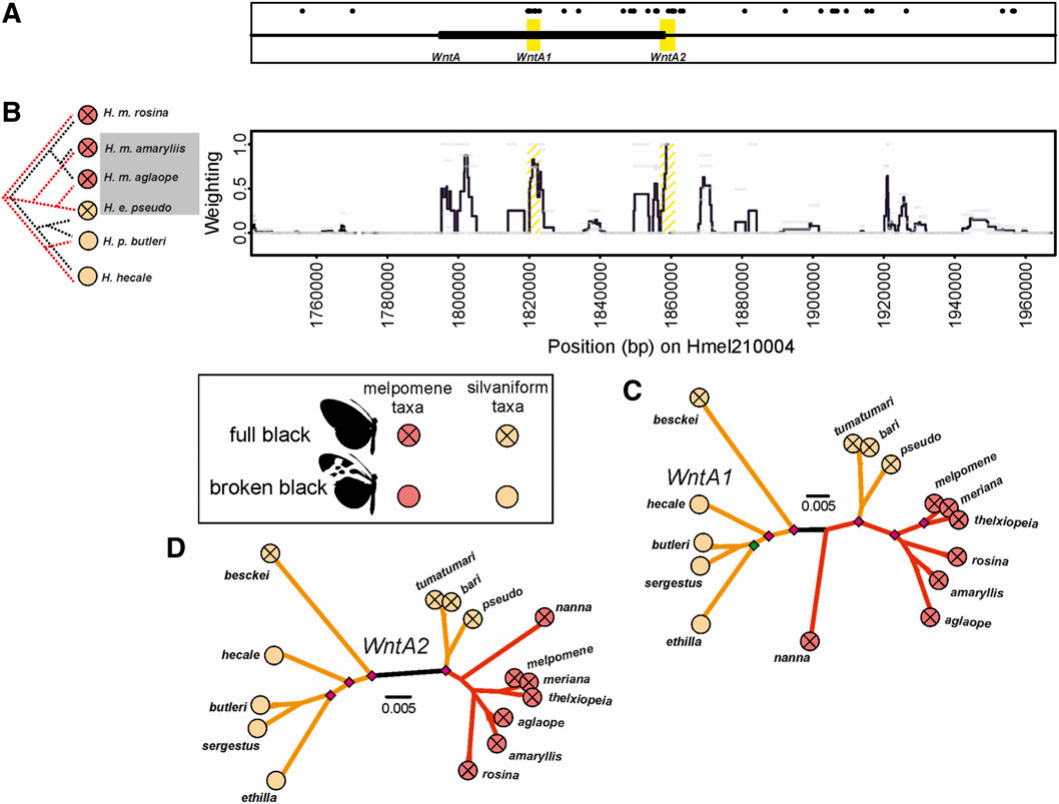
Paired phylogenetic Twisst comparison across the *WntA* region of scaffold Hmel210004. (A) Shows location of *WntA*, and putative functional elements. Black bar shows the *WntA* gene, yellow shading shows putative regulatory elements. Diagnostic fixed SNPS between phenotypes shown with black dots in (A). (B) Twisst comparison (100 SNP windows sliding by 25 SNPs) with Peruvian hybrid zone taxa. Mimetic phenotypes for taxa are shown by circles; red circles for *H. melpomene* clade and orange circles for silvaniform taxa. (C and D) Maximum likelihood phylogenies of putative elements; see Figure 2 legend for more detailed explanations. Note positions of black branches on phylogenies which have been drawn on the longest branches from where *H. besckei* connects to phylogeny.

**Figure 6:**
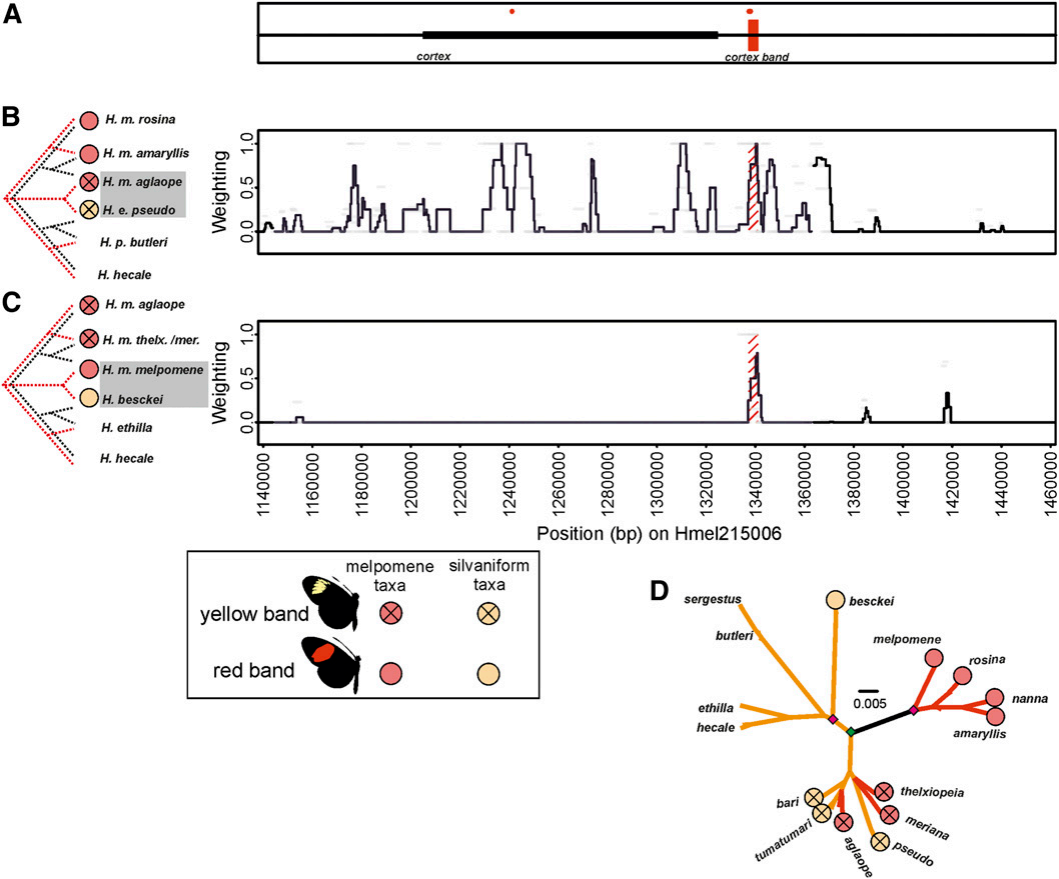
Paired phylogenetic Twisst comparisons across the *cortex* region of scaffold Hmel215006. (A) Shows location of *cortex*, and putative *cortex band* element. Black bar shows *Cortex* gene, red shading shows putative regulatory element. Diagnostic fixed SNPS between phenotypes shown with red dots in (A). Twisst comparison (100 SNP windows sliding by 25 SNPs) using (B) Peruvian hybrid zone taxa and (C) *H. besckei* and Guianese hybrid zone taxa. Mimetic phenotypes for taxa are shown by circles; red circles for *H. melpomene* clade and orange circles for silvaniform taxa. (D) Maximum likelihood phylogeny of the putative *cortex band* element; see Figure 2 legend for more detailed explanations.

For each Twisst comparison, the six taxa comprised three *H. melpomene* races and three silvaniform species (see Figure 2, Figure 3, Figure 4, Figure 5, Figure 6, and Table S7 for taxa used in each comparison). In each comparison, two of the *H. melpomene* taxa are from either side of a hybrid zone across which at least one phenotype of interest differs (*e.g.*, rays *vs.* no-rays). Across these hybrid zones, gene flow occurs freely along the genome except at regions controlling the phenotypic differences (Nadeau *et al.* 2012; Martin *et al.* 2013). The third *H. melpomene* taxon came from a geographically distant area, and while being genetically distinct from both, always shared the phenotype of one of the two hybrid zone *H. melpomene* (*e.g.*, no-rays). A silvaniform species (*H. elevatus* or *H. besckei*) was included in each comparison that shares the phenotype of interest (*e.g.*, rays) with one of the hybrid zone *H. melpomene*. The other two silvaniform species included *H. ethilla*, *H. hecale*, or *H. pardalinus*.

For comparisons with Guianese taxa, *H. ethilla* and *H. hecale* were used alongside Guianese *H. elevatus* taxa. However, for the Peruvian hybrid zone comparisons, *H. ethilla* and *H. pardalinus butleri* were used alongside *H. e. pseudocupidineus*. *H. pardalinus butleri* and *H. e. pseudocupidineus* are very closely related to each other across most of the genome (*F_ST_* ∼0 across 95% of the genome) (Kryvokhyzha 2014), and so including these taxa together with races of *H. melpomene* provides the sensitivity to differentiate shared variation that is associated with the phenotype (introgression or ancestral polymorphism) from shared variation that is unrelated to the phenotype (ancestry). If the mimetic silvaniform and *H. melpomene* taxa cluster together without the nonmimetic *H. melpomene* and silvaniform taxa, this is suggestive of shared variation associated with phenotype. Such a pattern should only occur at narrow color pattern regions due to our careful choice of taxa.

For comparisons with *H. besckei*, there is no nonmimetic *H. melpomene* race forming a hybrid zone with *H. m. nanna* available. Using *H. m. nanna* in Twisst analyses with other *H. melpomene* taxa (*H. m. thelxiopeia* and *H. m. aglaope*) resulted in a noisy analysis due to divergence between the *H. melpomene* taxa. Therefore, we instead compared *H. besckei* to *H. m. melpomene* from the Guianese hybrid zone (alongside *H. m. thelxiopeia* and *H. m. aglaope*).

### Identifying SNPs associated with phenotypes

We also looked for SNPs on our three focal chromosomes that were “diagnostically fixed” between taxa in different phenotype groups (Table S8). We excluded *H. e. tumatumari* from the no-ray taxa grouping as it appears to have independently evolved its no-ray phenotype. We used the same SNPs as in the Twisst analysis, but with up to 20% of genotype calls across taxa allowed to be missing for each SNP. For *optix* phenotypes, genotype calls were required to be present in all *H. melpomene meriana* and *H. besckei* samples, as these taxa are essential in differentiating diagnostically fixed SNPs for rays—no-rays from those for dennis/no-band—no-dennis/band. At *WntA* we looked for SNPs “diagnostically fixed” between *H. pardalinus* in one group (with the silvaniform *WntA* phenotype) and all *H. melpomene*, *H. elevatus*, and *H. besckei* in the other group (with a nonsilvaniform *WntA* phenotype). In this *WntA* analysis, genotypes had to be present in all *H. butleri* and *H. besckei* samples. As one of these groups is only made up of a single taxon (*H. pardalinus*), the results of the *WntA* analysis were noisier.

### Phylogenetic reconstruction at putative functional elements

At each of the putative functional elements we identified, we constructed unrooted trees using all 53 samples to determine the ancestral origin of each element. We included both variable and invariant sites within the boundaries of each element as delineated in our previous analyses using Twisst and fixed differences. Bcftools was used to remove poor quality genotype calls, and mark genotypes as missing if minimum read depth was <5 or GQ < 20. In addition, we also constructed a phylogenetic tree to determine the overall species relationships using all sites from across chromosomes 1 and 2, which we expect to show the species phylogeny as they do not contain any of the main color loci. We used RAxML to build all trees, with the GTRCAT model and 100 maximum likelihood trees to find the best tree, followed by 1000 bootstrap pseudoreplicates.

### Conservation and homology

Functional elements controlling gene expression may be conserved across taxa. In order to identify whether there was sequence conservation across Lepidoptera and in particular with *H. erato* (a distant relative and mimic of *H. melpomene*) at the putative functional elements we have identified, we retrieved the genome sequences of nine additional species from Lepbase (http://ensembl.lepbase.org); *H. erato demophoon* v1, *Junonia coenia* v1.0, *Bicyclus anynana* v1.2, *Danaus plexippus* v3, *Papilio machoan* v1.0, *Papilio polytes* v1.0, *Pieris napi* v1.1, *Amyelois transitella* v1, and *Bombyx mori* GCA000151625.1. We then identified scaffolds corresponding to the *WntA*, *optix*, and *cortex* loci with BLAST (Altschul *et al.* 1990). Fine-scale sequence conservation between *H. melpomene* and *H. erato* was visualized using the Artemis Comparison Tool (Carver *et al.* 2005). We then calculated pairwise conservation between *H. melpomene* and each of the other species using mVISTA (Frazer *et al.* 2004), an mLAGAN (Brudno *et al.* 2003) alignment and a conservation cut-off of 70% sequence identity.

### Data availability

The authors state that all data necessary for confirming the conclusions presented in the article are represented fully within the article. All original raw sequence data files are available via the ENA (accession number PRJEB37067). Supplementary tables and figures referred to in the text are available via figshare: https://doi.org/10.25386/genetics.12911180.

## Results

To identify putative regulatory elements in the *H. melpomene*-silvaniform clade, we use Twisst analyses across multiple hybrid zones to find signals of shared ancestry relating to wing pattern phenotypes and looked for diagnostically fixed SNPs between taxon groupings with these different phenotypes. We look for these patterns around loci known to control particular patterns, and where existing data have previously delimited an interval, we looked within that region. We first refine the *rays* and *dennis* loci, finding two putative loci for each of these phenotypes. Furthermore, we identify a 1.5 kb locus that we putatively delimit as the *optix band* region, as well as two loci at *WntA* that are associated with the full black discal forewing phenotype that *H. elevatus* shares with these *H. melpomene* races rather than the broken black of its sister species *H. pardalinus*. In contrast, our comparison using taxa from the Guianese hybrid zone shows no regions of shared derived ancestry between the broken band and no-rays forms of *H. elevatus* and *H. melpomene*, suggesting both these phenotypes evolved independently in mimetic *H. melpomene* and *H. elevatus* in the Guianas. We also identify a putative locus near the *cortex* gene associated with the yellow band phenotype. Finally, we use phylogenetic analysis to infer the ancestral origins of these putative regulatory elements across the *H. melpomene*-silvaniform clade, uncovering a complex history of introgression with different evolutionary origins for the various regulatory elements.

### Broader patterns of shared ancestry

On chromosome 18, our analyses identified only one additional ∼4 kb peak of shared ancestry (weighting of +1) between comimics that was consistent across hybrid zones and that was not located in the vicinity of *optix*. This was positioned ∼30 Mb from *optix*; at ∼3,014,000 bp on chromosome 18; and ∼227,000 bp on Hmel218005 (Figures S9–S11) within the gene HMEL034250g1. As a tblastx search of this gene in the *Drosophila melanogaster* genome does not return any good hits we are unable to speculate about its function. No peaks of shared ancestry with a weighting of +1 were found outside of the vicinity of *WntA* on chromosome 10 (Figure S12), and no large peaks showing consistent shared ancestry between comimics across comparisons (Figures S13–S15) were found on chromosome 15 (which contains the *cortex* gene). Thus, we demonstrate the near absence of regions of consistent shared ancestry between comimics outside the proximity of *WntA*, *optix*, and *cortex* on chromosomes 10, 15, and 18, respectively.

### The *ray* loci

Inferred recombination breakpoints around shared haplotypes have previously been used to delimit a 37 kb *ray* region on chromosome 18, further narrowed to 25 kb using SNPs perfectly associated with the hindwing rays (Wallbank *et al.* 2016). Our Twisst comparisons allow us to define two separable loci within this region with different evolutionary histories, a ∼6 kb region (Hmel218003 773301–779400) we call *ray1* and another ∼22 kb region (Hmel218003 781427–803436) we call *ray2* (Figure 2). Across both regions, we see a pattern of shared ancestry between rayed *H. elevatus* and *H. melpomene* mimics from the Peruvian hybrid zone (Figure 2B). However, in our *H. besckei* comparison, we see shared ancestry only across *ray2* (Figure 2C). Our phylogeny at *ray2* (Figure 2F) also shows that both no-ray and rayed Guianese and Peruvian *H. elevatus* show shared ancestry with rayed *H. melpomene* taxa.

Our Twisst comparison using the Guianese taxa without rays does not show shared ancestry between no-ray *H. elevatus* and *H. melpomene* from the Guianas at either *ray1* or *ray2* (Figure 2D). This suggests that the Guianese no-ray phenotypes in *H. elevatus* and in *H. melpomene* have evolved independently and are not the result of shared ancestry, with the no-ray phenotype in *H. e. tumatumari* evolving after introgression of ray alleles. Other peaks of shared ancestry are also found in this comparison, some of these relate to dennis (Figure 3, B and C; peaks at 813370–814253 bp; 816686–819634 bp) and band (Figure 4, B and C; peak at 728308–729971) phenotypes found in both *H. m. meriana* and *H. e. tumatumari*. It is unclear what might cause the additional peaks; however, as these are not consistent across phenotypes/comparisons they do not contribute to the locations of putative functional elements.

Due to independent evolution of the no-ray phenotype, *H. e. tumatumari* was excluded from the “diagnostically fixed” SNP analysis as its inclusion in the no-ray group would wrongly remove all “diagnostically fixed” SNPs (Table S8). We find that *ray1* contains 5 “diagnostically fixed” SNPs while *ray2* contains 67. Our phylogenies inferred using all taxa (Figure 2, E and F) show that all rayed and no-ray *H. elevatus* and all rayed *H. melpomene* group together at both loci, but that the no-ray *H. besckei* groups with no-ray *H. melpomene* only at *ray2*. These phylogenies also suggest the alleles in rayed taxa at these loci derive from different clades. The *ray1* rayed allele appears to originate from the *H. melpomene* clade (Figure 2E) as rayed individuals at *ray1* are clustered within the melpomene samples. In contrast, the *ray2* rayed allele appears to originate from the silvaniform clade, as rayed individuals at *ray2* are clustered within the silvaniform samples (Figure 2F).

### The *dennis* loci

Previously an ∼11 kb *dennis* region had been defined (Wallbank *et al.* 2016). We narrow this region by including *H. e. tumatumari* and *H. besckei* in separate Twisst comparisons and comparing these to Guianese *H. melpomene* taxa. As *H. e. tumatumari* and *H. besckei* are separate species and allopatric to each other, we used separate comparisons so as not to introduce noise. We define our loci using “diagnostically fixed” SNPs between our two phenotype groups, and signals of shared ancestry that are both consistent across Twisst comparisons and within the previously delimited ∼11 kb *dennis* region from Wallbank *et al.* (2016). On this basis, we delimit ∼1 kb (Hmel218003 813370–814253) and ∼3 kb (Hmel218003 816686–819634) regions that we term *dennis1* and *dennis2*, respectively (Figure 3). We split these as a consequence of a dip in shared ancestry consistent across all our comparisons. Both regions show shared ancestry between *H. elevatus* and *H. melpomene* mimics with the dennis phenotype across both the Peruvian (*H. e. pseudocupidineus* and *H. m. aglaope*) and Guianese hybrid zones (*H. e. tumatumari/bari* and *H. m. meriana/thelxiope*) (Figure 3, B and C). Our diagnostic fixed SNP analysis found that the *dennis1* region contained 20 “diagnostically fixed” SNPs with the correct pattern for *dennis* (or *optix band* as all red band taxa lack the dennis phenotype and so cannot be separated), while *dennis2* contains 18 such SNPs. There were only 40 other “diagnostically fixed SNPs” across the whole of Chromosome 18, with nine of these coming from our putative *optix band* region as expected. However, some of these other SNPs are also clustered, 15 SNPs clustered just upstream (Hmel218003 822665–825712) but outside the *dennis* region defined by Wallbank *et al.* (2016) and therefore outside of our defined *dennis* region (Figure 3D), and eight SNPs were clustered at the peak at ∼227,000 bp on Hmel218005. Phylogenies allowed us to define two separate phenotypic groups: one containing all no-dennis *H. melpomene* and *H. besckei* and the other containing mimetic dennis *H. melpomene* and *H. elevatus*. This is consistent with Wallbank *et al.* (2016). As the dennis *H. melpomene* races are nested within the silvaniform clade, these phylogenies suggest that the dennis phenotype originated in the silvaniform clade (Figure 3, D and E) and introgressed into *H. melpomene*, while the no-dennis alleles appear to have introgressed from *H. melpomene* into *H. besckei*.

### The *optix band* locus

Although our taxa do not allow us to tell shared ancestry due to band phenotypes from shared ancestry due to dennis phenotypes, we postulate that an *optix band* element would most likely be outside of the *dennis* (or *ray*) regions (Wallbank *et al.* 2016). This is based on (i) the existence of *H. timareta* races that lack red bands and dennis phenotypes (while other taxa have dennis phenotypes with rays but lack red bands) (Giraldo *et al.* 2008), and (ii) specimens of *H. melpomene* from the Guianese hybrid zone that have both dennis and red band phenotypes together (rather than having only one of these as seen in the main Guianese *H. melpomene* taxa) (J.M. personal observation). We hypothesize that any putative *optix band* element should also show shared ancestry across both Twisst comparisons. This is because the red band silvaniform *H. besckei* should show shared ancestry with red band races of *H. melpomene*, while *H. elevatus* races that have a yellow band should show shared ancestry with *H. melpomene* races that also have this phenotype. Using shared ancestry and diagnostically fixed SNPs we identify a single 1.5-kb region (Hmel218003 728308–729971), close to the 5′ end of *optix* that we putatively delimit as the previously unidentified *optix band* region (Figure 4, A and B). This region spans the only consistent peak of shared ancestry (outside of the *dennis* and *ray* regions) across all hybrid zones and includes nine “diagnostically fixed” SNPs between mimetic red and yellow band taxa. Furthermore, the wider region around this 1.5 kb also contains windows that inconsistently show a signal of shared ancestry between mimetic red band taxa and mimetic yellow band taxa (shown as gray bars with weighting of +1 in Figure 4, B and C). However, because the red band is always found in the absence of *dennis* in our taxa (and vice-versa), it should be noted that we cannot rule out that this region is in fact involved in the control of the *dennis* phenotype, or that the *dennis* regions are not involved in the control of the red band phenotype. The phylogeny at this locus shows that *H. besckei* is nested within the clade containing the red band *H. melpomene* races. While *H. m. aglaope*, *H. m. meriana* and *H. m. thelxiopeia* which all lack the red forewing band group with the other silvaniform species (Figure 4D), suggesting an ancestral silvaniform clade origin for the allele causing loss of the red band.

### Two *WntA* loci

Around *WntA* we identify two separate loci that we term *WntA1* (Hmel210004 1819577–1822844) and *WntA2* (Hmel210004 1857021–1860913), which show a signal of shared ancestry between *H. melpomene aglaope*/*amaryllis* and *H. elevatus pseudocupidineus*, but not between *H. melpomene aglaope*/*amaryllis* and *H. pardalinus butleri* (Figure 5B). This pattern is consistent with what we would expect in regions involved in controlling the full black discal forewing phenotype that *H. elevatus* shares with these *H. melpomene* races (and which replaces the broken black patterning found in *H. pardalinus* and other silvaniforms in this part of the wing). These two loci were the only peaks also supported by the results from our “diagnostically fixed” SNP analysis between *H. pardalinus butleri* and all taxa with a *H. melpomene* type phenotype (all *H. melpomene* and *H. elevatus* races and our *H. besckei*). In total *WntA1* and *WntA2* contained, respectively, 12 and 6 of the 252 “diagnostically fixed” SNPs on chromosome 10. Thus, *WntA1* and *WntA2* together contain 7% of these “diagnostically fixed” SNPs in just 0.04% of the length of the chromosome.

The phylogenies at *WntA1* and *WntA2* clearly show *H. elevatus* races grouping within (*WntA1*) or close to (*WntA2*) the *H. melpomene* clade. In both phylogenies, *H. besckei* was found outside the rest of the silvaniforms (excluding *H. elevatus*), rather than grouping with *H. ethilla* as it does in the species tree (Figure S16). This suggests that there may be some shared ancestry in these regions between *H. besckei* and the other *H. melpomene* races. Both phylogenies suggest that the full black discal forewing phenotype which replaces broken black in *H. e. pseudocupidineus* originates from the *H. melpomene* clade. In contrast to our results with Peruvian taxa, our comparison using taxa from the Guianese hybrid zone (Figure S17) shows no regions of shared derived ancestry on chromosome 10 between the broken band forms of *H. elevatus* (*H. e. tumatumari* and *H. e. bari*) and *H. melpomene* in the Guianas (*H. m. meriana* and *H. m. thelxiopeia*), relative to *H. m. melpomene*, which lacks the broken band phenotype. Given previous research has shown that this phenotype is controlled by *WntA* in *H. melpomene* (Morris *et al.* 2019), our results here suggests that just like the no-rays phenotype, the broken band phenotype in the Guianas also evolved independently in mimetic *H. melpomene* and *H. elevatus*.

### The cortex band locus

Only the yellow forewing band phenotype (and not the yellow hindwing bar) is found in both our Peruvian and Guianese hybrid zone taxa. We identified a ∼3.5 kb region near *cortex* (Hmel215006 1337470–1340886) that shows shared derived ancestry between yellow band *H. e. pseudocupidineus* and *H. m. aglaope* from the Peruvian hybrid zone (Figure 6B) and between red band *H. besckei* and *H. m. melpomene* (Figure 6C). This region also contained two of the three “diagnostically fixed” SNPs on chromosome 15, between red band *H. melpomene* and *H. besckei* and our yellow band *H. melpomene* and *H. elevatus*. These results suggest that this region may be involved in controlling the yellow band phenotype. We term this locus “*cortex band*.” Phylogenetic reconstruction at *cortex band*, shows that this region is also shared between all yellow band *H. elevatus* and *H. melpomene* taxa (Figure 6D), and that the putative yellow band allele appears to have originated in the silvaniform clade before then introgressing into the *H. melpomene* taxa with yellow bands. However, *H. besckei* was not found on the branch with red band *H. melpomene*. Therefore, the signal of shared ancestry seen between *H. besckei* and red band *H. melpomene* in the Twisst comparison may not be due to shared ancestry between these two taxa in an allele that results in a lack of yellow band. Instead it may be an artifact of the shared ancestry between yellow band *H. melpomene* races and *H. elevatus*, which would make the *H. melpomene* haplotype in these races more closely related to *H. hecale* and *H. ethilla* haplotype than that in *H. besckei*. However, given that we only detect this phylogenetic signal at this region, and the location of our diagnostic fixed differences, this single peak is still our best candidate for a “*cortex band*” element, even if we cannot rule out other regions that we miss due to complex pleiotropic interactions that do not give such an expected phylogenetic signal of shared ancestry.

### Modular conservation across *Heliconius* and the Lepidoptera

**Figure 7:**
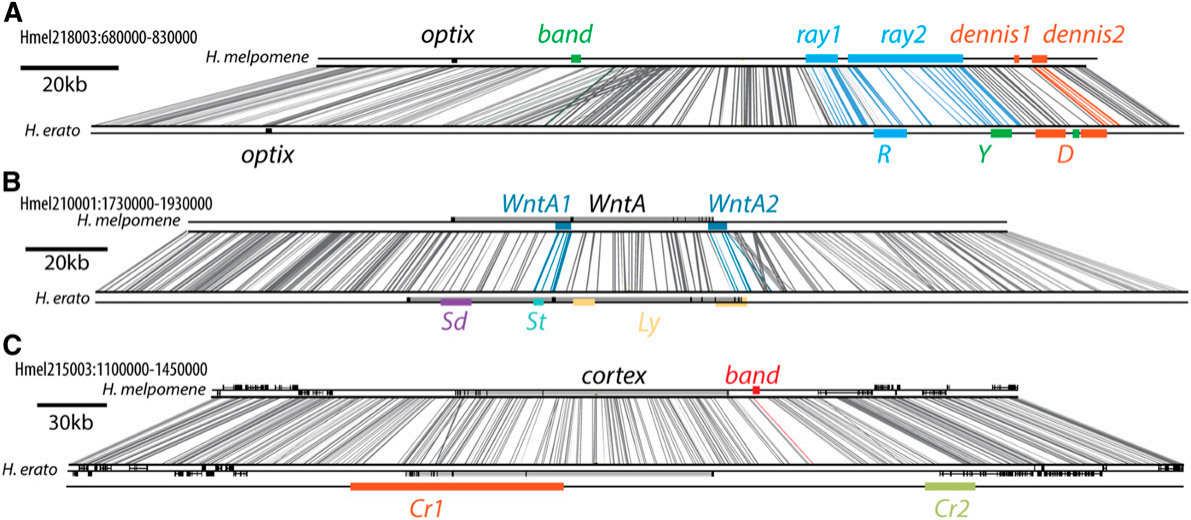
ACT-BLAST alignment of three color pattern regions between *H. melpomene* and its comimic *H. erato*. In each panel, the *H. melpomene* scaffold (top) is compared to the *H. erato* scaffold (bottom) with each BLAST hit connected by a line. *H. melpomene* is annotated with elements identified herein, and *H. erato* is annotated with elements controlling convergent phenotypes as identified in Van Belleghem *et al.* (2017). In (A) one of the *H. melpomene* elements associated with the ray pattern element (blue; *ray2* in *H. melpomene*; *R* in *H. erato*) contains homologous sequence with the ray pattern element in *H. erato*, as indicated by the connecting colored lines. As does one of the elements associated with dennis pattern in *H. melpomene* and the *H. erato* dennis associated element (orange; *dennis2* in *H. melpomene*; *D* in *H. erato*) (Table S20). The elements associated with band (green; band in *H. melpomene*; *Y* in *H. erato*) are in distant positions, but *H. erato Y* is situated in conserved sequence which is included in the *H. melpomene ray2* element. In (B) the *H. melpomene WntA1* and *WntA2* elements both contain homologous sequence to the *H. erato* elements *St* and *Ly* (Table S20). In (C) *band* is shown to not be homologous to either of the previously identified elements (*Cr1* and *Cr2*) in *H. erato*. *H. erato* elements are colored based on scheme used in Van Belleghem *et al.* (2017).

Fine-scale sequence conservation between *H. melpomene* and *H. erato* at and around *optix*, *WntA*, and *cortex* is shown in Figure 7. As expected, we find that these two genomes are largely colinear, with large sections of homology across the genomes. *Ray1* contains a substantial amount of conserved sequence between the *H. melpomene* and *H. erato*, as well as a 2000 bp region (Hmel218003:778000–780000; Figure S18) deeply conserved across the Lepidoptera, indicating the presence of a conserved functional element within *ray1* of both species (See Table S19 for scaffold locations in other Lepidoptera). Our analysis also finds narrow regions of homology (Table S20) between the *ray2* locus in *H. melpomene* and the *R* locus controlling the ray phenotype in *H. erato* (Van Belleghem *et al.* 2017). The *H. melpomene dennis1* element has very low sequence conservation with *H. erato* and no conserved sequence with other Lepidoptera. However, the *H. melpomene dennis2* element contains extensive sequence conservation with *H. erato* (as well as a short region of conservation with other nymphalids; Hmel218003: 819500–82000), and we also find a narrow region of homology between *dennis2* (Table S20) and the *D* locus controlling the dennis phenotype in *H. erato*. The *H. melpomene optix band* element identified here is located within 50 kb of the gene *optix*, but at a substantial distance from the Y element that controls the corresponding phenotype in the *H. erato* clade (Van Belleghem *et al.* 2017) in a region of low conservation with *H. erato*. The physical distance between the elements in the two species indicates that the evolution of the red band occurred by evolution of regulatory changes at unrelated loci. At the *WntA* locus, the *WntA1* and *WntA2* elements we identify contain homologous sequence with the *H. erato St* (a likely promoter of the *WntA* gene) and *Ly* elements respectively (Table S20). The *WntA1* element contains two peaks of conservation with multiple other lepidopteran species (Figure S18), corresponding to the two 3′ coding exons of the *WntA* gene. At *cortex*, the *H. melpomene cortex band* element was located between the two large yellow bar-linked regions identified in *H. erato* (Van Belleghem *et al.* 2017). Overall we find that several of the putative regulatory elements we have identified as controlling patterning in *H. melpomene* and the silvaniform clade (*ray2*, *dennis2*, *WntA1*, and *WntA2*) appear to be at least partly homologous with regulatory elements proposed to control similar phenotypes in *H. erato*, a species from which they diverged ∼12 MYA (Kozak *et al.* 2015).

## Discussion

In this study, we have investigated the contributions of three genetic modes of evolution (divergent genetic mechanisms, parallel genetic evolution, and collateral evolution) to explain convergent wing color pattern phenotypes found in *Heliconius* butterflies (*H. melpomene*, *H. elevatus*, *H. besckei*, and *H. erato*). Using phylogenetic analyses, we have identified strong and narrow signals of shared ancestry related to wing pattern phenotypes in *H. melpomene*, *H. elevatus*, and *H. besckei* around all three main color pattern genes (*optix*, *WntA*, and *cortex*). This is indicative of collateral evolution of putative regulatory elements among these closely related species. In contrast, signals of consistent shared ancestry among these taxa were low outside of these regions. However, we also find that convergent phenotypes between *H. melpomene* and *H. elevatus* in the Guianas appear to have arisen independently and so are not a result of collateral evolution. We also show that four out of the seven putative regulatory elements around *optix* and *WntA* in *H. melpomene* also show some homology to regulatory elements controlling similar phenotypes in its distant comimic *H. erato*. Thus, convergent phenotypes between these more distantly related species appear to result from a combination of convergent parallel evolution and divergent genetic mechanisms. Overall, our results show that all three genetic modes of evolution underlie convergent phenotypes among mimetic *Heliconius* species, but that these likely operate at different evolutionary timescales.

### Modularity of mimicry facilitates pattern switching

Recent studies in a variety of organisms have demonstrated the importance of combinatorial evolution, where ancient alleles are reused in novel combinations to generate new phenotypes and adaptive combinations (Marques *et al.* 2019). This can lead to adaptive convergent changes more rapidly than evolution via divergent genetic mechanisms or parallel genetic evolution. This can be seen in cichlids, where regulatory changes at the gene *agouti-related peptide 2* are associated with the convergent stripe evolution across species (Kratochwil *et al.* 2018), and in sticklebacks, where recurrent deletions of the same *pitx1* enhancer in different populations have led to reduced or lost pelvic structures (Chan *et al.* 2010). Another excellent example are the diverse wing patterns of *Heliconius* butterflies. These butterflies appear to have a flexible toolkit of *cis*-regulatory enhancers (Wallbank *et al.* 2016; Van Belleghem *et al.* 2017) through which gene expression changes can rapidly alter phenotypes and drive adaptive evolution (Wray 2007), with a single mutation at an enhancer potentially enough to have major phenotypic effects (Chan *et al.* 2010; Frankel *et al.* 2012). Such genetic architecture combined with introgression can facilitate adaptive evolution through the swapping of these enhancers among lineages of *Heliconius* (Wallbank *et al.* 2016; Moest *et al.* 2019; Lewis and Van Belleghem 2020). For example, the evidence suggests that the ancestral sources of the *ray* and *dennis* elements were different, with the rays phenotype originating in the *H. melpomene* clade and the dennis phenotype originating in the silvaniform clade, before being brought together as the dennis-rayed phenotype in both *H. melpomene* and *H. elevatus* (Wallbank *et al.* 2016).

**Figure 8:**
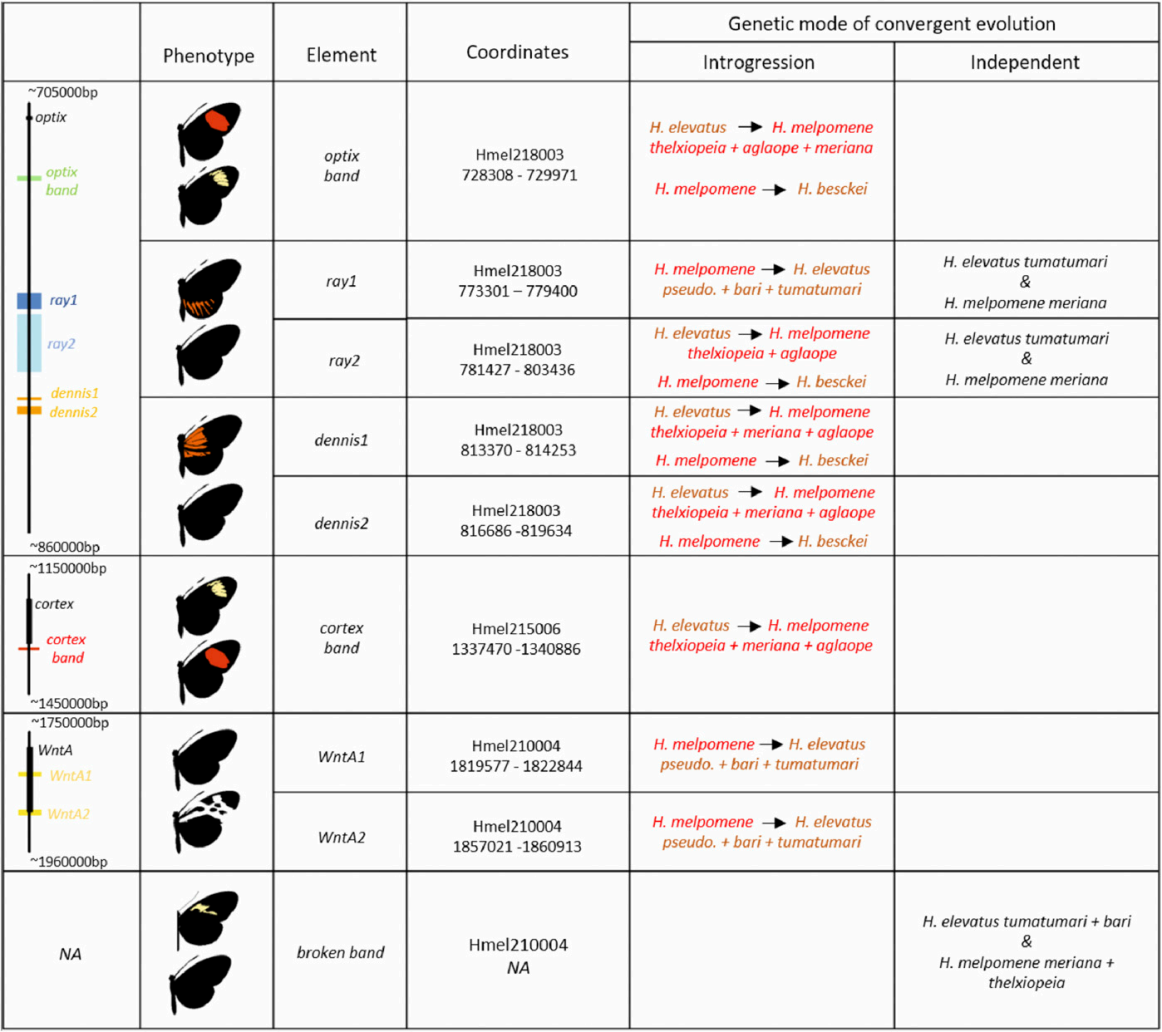
A summary of the locations of putative *cis*-regulatory elements reported here, and the genetic mode of evolution causing mimetic convergence between *H. melpomene* races and silvaniform species at each element. In the introgression column: red to orange names indicate introgression from *H. melpomene* (*H. melpomene* clade origin) into silvaniform taxa, while orange to red names indicate introgression from silvaniform taxa (silvaniform clade origin) into *H. melpomene*. There is no identified element for *broken band* due to a lack of shared ancestry and its independent evolution in each species. The schematic in the left most column shows the locations of each element (colored labels) with respect to neighboring genes (black labels).

Our analysis, which narrows the *dennis* and *ray* elements and splits them into two loci each, is consistent with this finding of multiple origins, even finding separate origins for each *ray* locus. We also identify a putative *optix band* locus near *optix* that suggests the red band is ancestral to the *H. melpomene* clade, while its absence is ancestral to the silvaniform clade. The two putative loci near *WntA* that we propose control the full black melanic discal forewing phenotype of *H. elevatus* (which replaces the broken black pattern in other silvaniforms) also appear to be ancestral to the *H. melpomene* clade. Finally, our putative *cortex band* loci suggests that the yellow band phenotype was ancestral to the silvaniforms. By expanding on the range of taxa and loci considered in previous studies, our results paint a picture of multiple loci originating in different clades and then being brought together via introgression to derive the Amazonian *Heliconius* races that we see today. For example, around *optix*, it appears that the *ray2* rays allele, the *dennis1* and *dennis2* dennis alleles, and the *optix band* no-band allele originated in the silvaniform clade, and, through gene flow with *H. elevatus*, introgressed into *H. melpomene*. In contrast, the *ray1* ray allele introgressed from *H. melpomene* into *H. elevatus*, while the *optix band* red band allele, *dennis1* and (possibly) *dennis2* no-dennis alleles, and the *ray1* no-ray allele introgressed from *H. melpomene* into *H. besckei* (summarized in Figure 8). These loci may all have introgressed separately, alternatively for example, the two dennis loci may have introgressed together, perhaps even along with *ray2* and *optix band* as a single haplotype, and then subsequently been broken up by recombination. Reconstructing the exact evolutionary history with the order and timing of introgression events at these narrow regions may prove to be difficult.

Although modularity is just one hypothetical mode by which these putative regulatory elements may impact on wing color patterns, our results are currently the most compelling evidence for modularity in the *cis*-regulatory architecture of *Heliconius* wing pattern variation. Our work therefore adds to a growing body of evidence for a profound role of modularity and introgression in the evolution of mimicry in *Heliconius* butterflies. Furthermore, our results, along with examples such as pesticide resistance in mice and *Helicoverpa* moths (Song *et al.* 2011; Valencia-Montoya *et al.* 2020), beak adaptations in Darwin’s finches (Grant and Grant 2010; Lamichhaney *et al.* 2015), vectorial capacity in mosquitoes (Fontaine *et al.* 2015), and human evolution (Abi-Rached *et al.* 2011; Huerta-Sánchez *et al.* 2014; Sankararaman *et al.* 2016) highlight the potential for introgression to act as an adaptive facilitator.

### Independent evolution driving mimetic convergence in the Guianas

Our analyses have allowed us to putatively identify regulatory elements shared between *H. melpomene* and silvaniform taxa, with evidence suggesting that this has occurred via introgression (Dasmahapatra *et al.* 2012; Pardo-Diaz *et al.* 2012; Wallbank *et al.* 2016; Enciso-Romero *et al.* 2017). However, although introgression is one way in which major *cis*-regulatory changes to expression can occur, changes can also occur via *de novo* evolution of *cis*-regulatory enhancers (Wray 2007). This can occur via sequence duplications (Eichenlaub and Ettwiller 2011), transposable elements that lead to the translocation of regulatory elements from one gene to another (Daborn 2002; Domené *et al.* 2013), or the co-option of existing regulatory sequences to derive novel expression patterns (Rebeiz *et al.* 2011). While our analyses cannot identify the elements that are not shared across taxa, we are able to show that the convergent no-ray phenotypes in mimetic *H. melpomene meriana* and *H. elevatus tumatumari* appears to have resulted from independent evolution within each species because there is no signal of shared ancestry at the *ray1* or *ray2* regions between these taxa (Figure 2, E and F). Given our evidence that introgression of phenotypes between *H. melpomene* and silvaniform taxa appears to have occurred in Peru, and that *H. elevatus tumatumari* appears to have the rayed allele found in other rayed races of *H. melpomene* and *H. elevatus*, we hypothesize that *H. elevatus tumatumari* has secondarily and independently evolved a no-ray phenotype. In contrast, the lack of rays in *H. melpomene meriana* appears to be due to having the no-ray allele found in other *H. melpomene* races (Figure 2, E and F). This lack of rays may either be the ancestral state in *H. melpomene*, with *H. melpomene meriana* then later gaining the dennis and yellow band phenotypes through introgression, or alternatively, a result of the ray allele of *H. melpomene meriana* being replaced by a no-ray allele through recombination with *H. melpomene melpomene*. In addition, our analyses also show that the forewing broken band phenotype found both in *H. melpomene meriana/thelxiopeia* and *H. elevatus tumatumari/bari* in the Guianas must also have independent origins as we see no signal of shared ancestry between these taxa around *WntA*.

Our results indicate that mimicry via introgression between *H. elevatus* and *H. melpomene* has therefore not occurred consistently across their ranges. A possible scenario is that introgression first occurred in Peru allowing species to switch or perhaps create new mimicry rings, with these newly introgressed alleles and then simply persisting in the Guianas, where independent convergent evolution has refined local mimicry leading to *H. elevatus* losing the rays and melanic *WntA* phenotypes. This means that both introgression and the independent convergent evolution of novel *cis*-regulatory elements, has been important in driving mimicry between these two species.

### Convergence and sequence conservation

Our results further support the notion that *cis*-regulatory modularity is common across mimicry genes in *Heliconius*. Having refined and identified putative *cis*-regulatory elements, we investigated whether these intervals showed sequence conservation between *H. erato*, *H. melpomene* and other Lepidoptera, using sequence conservation as a proxy for *cis*-regulatory function. While the *band* element regulates the expression of *optix* in both *H. erato* and *H. melpomene*, this was achieved by divergent genetic mechanisms. On the other hand, we have found evidence of parallel evolution in the modification to the 5′ noncoding region of *WntA* in both *H. melpomene* and *H. erato*; these modifications occurred in evidently homologous regulatory elements, despite their independent evolution.

The broad region around the *ray* and the *dennis* elements contains a high density of deeply conserved sequences, but appears to be a hotspot for the modification by selection within *Heliconius*. Likewise, repeated modifications in the 5′ promoter region of *WntA* could indicate a role for this region as a hotspot for modification by selection. Possible explanations for the repeated reuse of the same noncoding regions in regulatory evolution focus on aspects of the structure and function of ancestral *cis*-regulatory elements. Aspects such as low pleiotropy, and the greater variety of mutations that can cause functional changes, makes these regions predisposed toward gaining new regulatory functions (Stern 2013). Potentiating mutations could arise stochastically and neutrally in nonfunctional sequence, or they could occur in pre-existing regulatory elements, which already have the structure and function necessary to act as a regulator (Blount *et al.* 2008). Modification of a site with ancestrally shared potentiating mutations would require fewer mutational steps as opposed to *de novo* generation of a regulatory element, increasing the probability that the novel function will evolve at that site; *i.e.*, pre-adaptation, as was observed in the evolution of citrate metabolism in populations of *Escherichia coli* (Blount *et al.* 2008). If potentiating mutations were present in the ancestor, then we would expect to find regulatory mutations to occur close together in convergent species, and indeed, we observed this in the *dennis2*, *ray2*, *WntA1*, and *WntA2* elements. There are still only a few well-studied examples of independent convergence in regulatory sequence (Booker *et al.* 2016; Partha *et al.* 2017; Kratochwil *et al.* 2018; Tollis *et al.* 2018; Feigin *et al.* 2019) as not many *cis*-regulatory mutations that pertain to convergent phenotypes have yet been identified. It is therefore yet to be seen whether there is a general trend of convergence across taxa at this level of granularity, but, as more examples are characterized, whether or not such a trend persists will be revealed.
